# Numerical Investigation and Multi-Objective Optimization on Forming Quality of CFRP/Al Self-Piercing Riveting Joint

**DOI:** 10.3390/ma18061233

**Published:** 2025-03-11

**Authors:** Feng Xiong, Xuehou Yu, Shuai Zhang, Dengfeng Wang, Hongyu Xu

**Affiliations:** 1 Joint International Research Laboratory of Intelligent Manufacturing & Control of Key Parts for Energy-Efficient & New Energy Vehicles, Ministry of Education, Chongqing University of Technology, Chongqing 400054, China; 2 State Key Laboratory of Automotive Simulation and Control, Jilin University, Changchun 130022, China; 3 State Key Laboratory of Intelligent Green Vehicle and Mobility, Tsinghua University, Beijing 100084, China; 4 School of Vehicle and Traffic Engineering, Henan University of Science and Technology, Luoyang 471003, China

**Keywords:** CFRP/Al, self-piercing riveting, forming quality, multi-objective optimization, Taguchi–TOPSIS, multi-material joint

## Abstract

Self-piercing riveting (SPR) has become a highly promising new method for connecting dissimilar materials in multi-material vehicle bodies, while the joint’s forming quality which largely affects its connection performance lacks sufficient research. This study conducted a detailed numerical investigation on the forming quality of carbon-fiber-reinforced polymer (CFRP)/aluminum alloy (Al) SPR joint and proposed a novel multi-objective optimization strategy. First, the finite element (FE) model of CFRP/Al SPR joint forming was established and then verified to monitor the forming process. Second, based on FE numerical simulation, the action laws of rivet length and die structural parameters (die depth, die gap, and die radius) on the joint’s forming quality indicators (bottom thickness and interlock value) were systematically studied to reveal the joint’s forming characteristics. Finally, taking the rivet length and die structural parameters as design variables and the above forming quality indicators as optimization objectives, a hybrid Taguchi–Technique for Order Preference by Similarity to Ideal Solution (TOPSIS) method was proposed to conduct the multi-objective optimization of the joint’s forming quality. According to the outcomes, the bottom thickness and interlock value of the joint were respectively increased by 10.18% and 34.17% compared with the baseline design, achieving a good multi-objective optimization of the joint’s forming quality, which provides an effective new method for efficiently predicting and improving the forming quality of the CFRP/Al SPR joint.

## 1. Introduction

**Figure 1:**
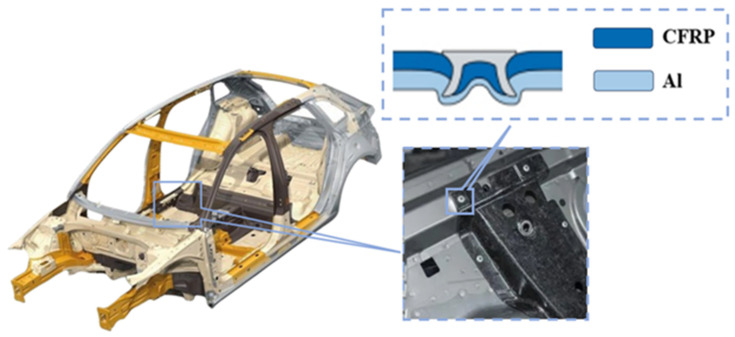
Example of SPR joint in CFRP/Al connections of a vehicle body.

Light weight of a vehicle body is vital for improving the vehicle energy efficiency, as reducing body weight can boost the range of electric vehicles or improve the fuel economy of traditional fuel vehicles. However, it may also lead to a decrease in body structural strength, posing a threat to occupants’ safety in the event of a collision. To balance these requirements, the multi-material lightweight vehicle body composed of various materials, such as aluminum alloy (Al), carbon-fiber-reinforced polymer (CFRP), etc., has become a hot topic and focal direction of current research [1,2]. Nevertheless, the connection between heterogeneous materials in multi-material vehicle bodies, especially CFRP and Al, remains a current technical difficulty. Self-piercing riveting (SPR) has become a promising solution and been preliminarily applied in CFRP/Al connections of a vehicle body, as shown in Figure 1, due to its high forming efficiency [3], simple process [4], and good connection performance. However, the forming quality of the CFRP/Al SPR joint, which largely affects the joint’s connection performance, lacks sufficient research present to now.

To clarify the forming characteristics of SPR joint, identify the factors affecting its forming quality and develop optimization methods, extensive research had been conducted by numerous scholars. Rezwanul Haq [5] studied the cross section of SPR joints, identifying several parameters that influence the forming quality of SPR joints, and discussed effective techniques to improve the joint’s riveting quality. Karathanasopoulos et al. [6] analyzed the effects of two geometric parameters (inner radius of the rivet leg and central tip depth of the die) on two critical process quality indicators of the joint. Ma et al. [7] studied the effects of rivets and dies of different sizes on the steel-Al dissimilar material SPR joints, providing insights into combined optimization of rivet and die to improve the joint’s quality. Wang et al. [8] investigated SPR joints between steel and Al sheets using experiments and simulations to analyze the effects of rivet length, edge angle, shank thickness, die diameter, and die depth on the joint’s forming quality indicators. Satoshi Achira et al. [9] used finite element simulation to investigate the SPR of ultra-high strength steel and Al sheets, focusing on the impact of sheet morphology on the joint’s forming quality. Zhou et al. [10] investigated the influence of process parameters on the formability, static tensile properties, and fatigue performance of SPR joints between Al and high-strength steel. Du et al. [11] investigated the SPR joint of aluminium-steel hybrid sheets by developing a 2D axisymmetric numerical model to study the effects of material strength and sheet thickness on the process quality. Liu et al. [12] studied the joining and damage mechanisms of SPR joints between CFRP and Al, proposing a shear-based composite damage model and numerically simulating the SPR forming process to analyze damage evolution. Wang et al. [13] proposed a post-curing SPR (PC-SPR) method to connect CFRP with Al sheets, and investigated the influence of CFRP sheet thickness and joint’s structure on the riveting quality. Namsu Park et al. [14] studied multi-material, multi-layer SPR connections, validating the finite element modeling method with experiments and demonstrating its high accuracy in predicting joint forming quality. Zhao et al. [15] systematically studied the effects of top plate thickness, bottom plate thickness, and rivet length on the forming mechanism of SPR joint through a combination of experiments and finite element modeling. Ang et al. [16] optimized the joint forming quality through process parameter adjustments and advanced technologies. Zhang et al. [17] optimized the forming quality of self-piercing riveted joints using a quadratic polynomial response surface model combined with the NSGA-II algorithm, and validated the effectiveness of this method through cross-sectional measurements. Kong et al. [18] analyzed the effects of structural parameters on the forming quality and mechanical properties of CFRP/Al SPR joint, and conducted the multi-objective optimization using GRA coupled with the entropy weight method. Li et al. [19] proposed a multi-fidelity optimization framework for SPR joint forming, incorporating an improved Latin Hypercube Sampling method and a transfer-learning-based neural network surrogate model, etc.

However, according to the existing relevant research, the following findings can be summarized: (1) The vast majority of current research on the forming characteristics and quality of SPR joint is related to different metals, while there are relatively few reports on that between heterogeneous materials like composite materials and metals, especially CFRP and Al. Considering the vastly different properties of CFRP and Al, there is an urgent need for in-depth research on the forming characteristics and quality of CFRP/Al SPR joint. (2) Most of the current research related to the forming characteristics and quality of SPR joint mainly relies on forming experiments, which is time-consuming, laborious and of poor repeatability. In contrast, Finite-element (FE) model based numerical simulation are confirmed as accurate and efficient method for handling such issues, while few nu-merical studies have been reported concerning forming characteristics and quality of CFRP/AL SPR joint at present. To monitor the forming process more efficiently and better reveal the forming characteristics, it is necessary to further develop the FE numerical simulation method for CFRP/AL SPR joint. (3) Currently there are relatively few studies reported on the optimization of forming quality of SPR joint, and most of which adopts optimization methods based on approximate models, which rely on substantial experimental samples to achieve required predicting accuracy and thus is much time-consuming. Considering the simulation time of CFRP/Al SPR joint forming is relatively long, it is necessary to explore more efficient methods for optimizing the forming quality of CFRP/AL SPR joint.

**Figure 2:**
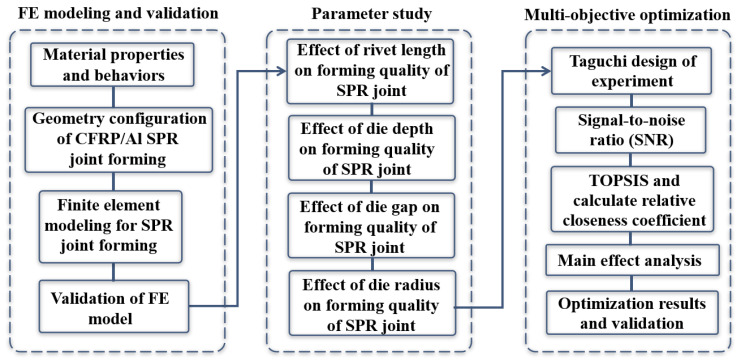
Flow chart of the present study.

Hence, in the present study, a detailed numerical investigation on the forming characteristics and quality of CFRP/Al SPR joint was conducted and a novel multi-objective optimization method based on hybrid Taguchi–TOPSIS was further proposed. Specifically, the FE model of CFRP/Al SPR joint forming was first established and then verified to monitor the forming process. Second, based on FE numerical simulation, the action laws of rivet length and die structural parameters (die depth, die gap, and die radius) on joint’s forming quality indicators (bottom thickness and interlock value) were systematically studied to reveal the joint’s forming characteristics. Finally, with the rivet length and die structural parameters taken as design variables and the above forming quality indicators as optimization objectives, a hybrid Taguchi–TOPSIS method was proposed to conduct the multi-objective optimization of the joint’s forming quality. The proposed optimization method employs Taguchi orthogonal experimental design to provide experimental samples and employ TOPSIS to explore the optimal design, which could reduce the computational cost significantly. Figure 2 outlines the flowchart of the present study.

## 2. Material Properties and Behaviors 

### 2.1. Aluminum Alloy(Al) Plate

**Figure 3:**
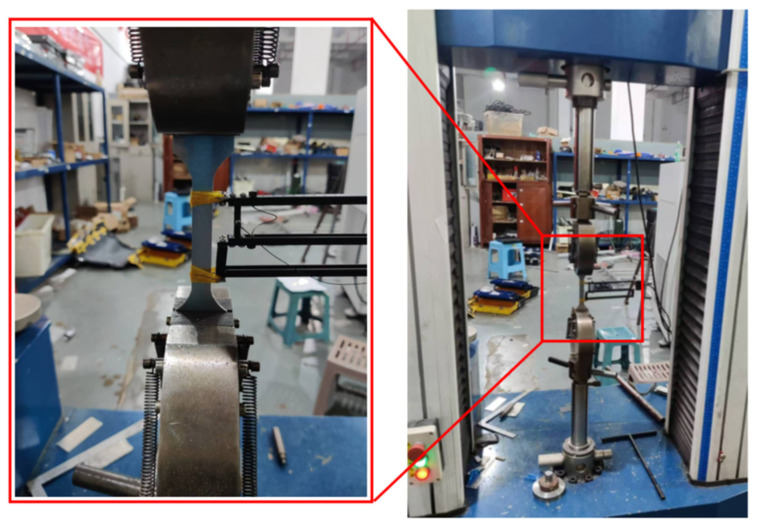
Quasi-static tensile test of Al.

**Figure 4:**
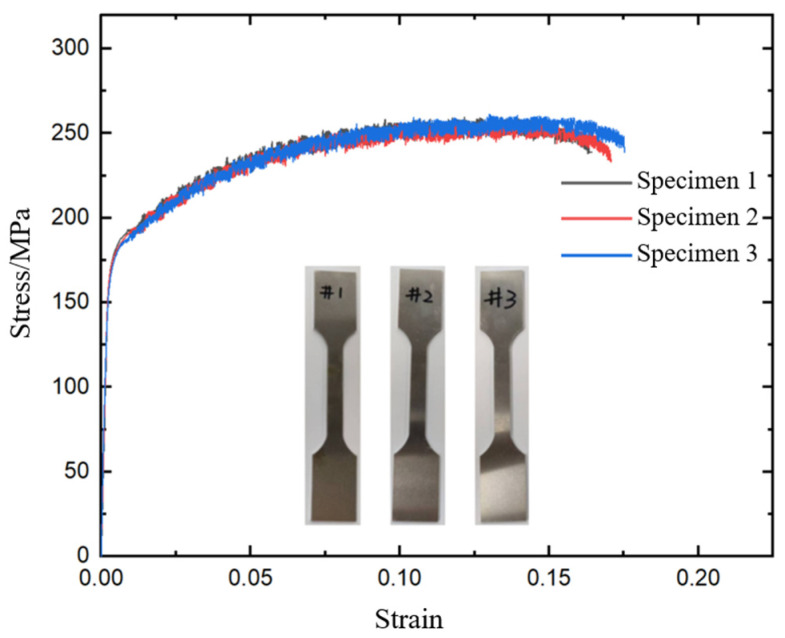
Stress–strain curves of Al.

In this study, the lower Al plate for CFRP/Al SPR joint forming is made of aluminum alloy, with material density ρ = 2700 kg/m^3^, Poisson’s ratio λ = 0.3. Figure 3 demonstrates the quasi-static tensile tests of Al through an electronic universal testing machine, and Figure 4 exhibits the stress–strain curves obtained. Three tensile tests were repeated to verify the validity of the experimental results. The mechanical behaviors of Al was simulated using an elastoplastic model in Abaqus.

### 2.2. Self-Piercing Rivet

**Figure 5:**
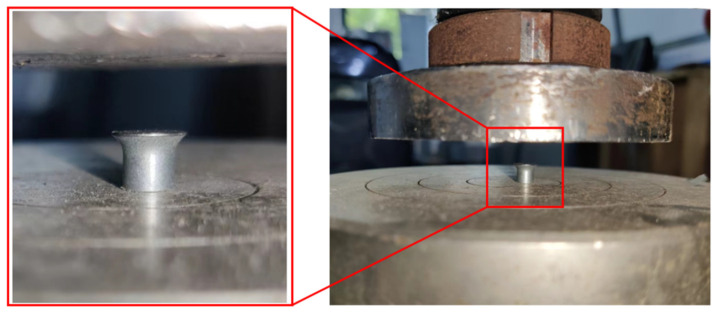
Compression test of self-piercing rivet.

**Figure 6:**
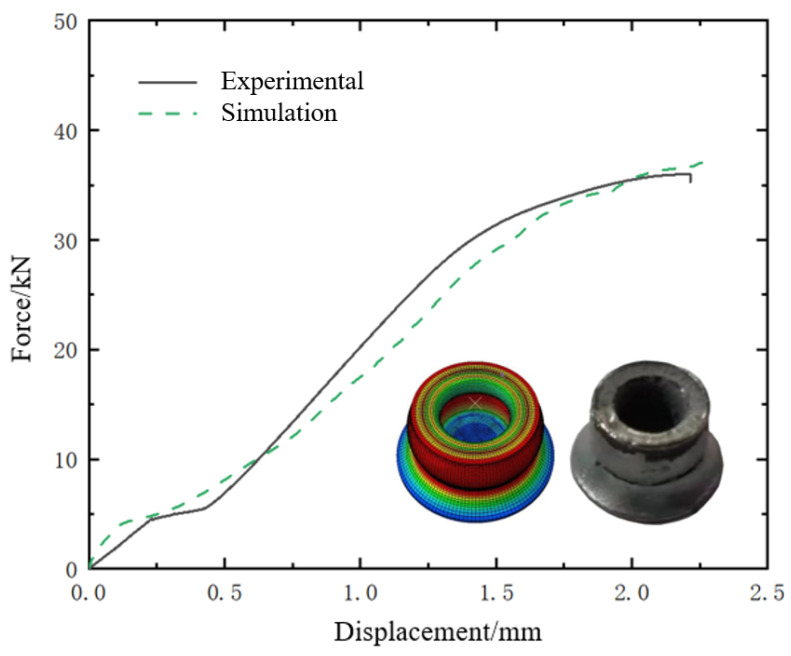
Comparison of force versus displacement curves between experiment and simulation.

The self-piercing rivet used in the process is made of 1045 high-strength steel, with a material density of 7850 kg/m^3^. Since its material properties could not be obtained from the manufacturer, a numerical simulation approach was employed to reverse-engineer the material behaviors. Specifically, to monitor the load conditions experienced by the rivet during the SPR joint forming process, a compression test was conducted on the rivet at a speed of 0.5 mm/min, as shown in Figure 5. Meanwhile, a FE model of the rivet under similar load conditions was established, for which the elastoplastic constitutive model was adopted to monitor the material behaviors of rivet. Figure 6 demonstrates the comparison of force-displacement curves and corresponding deformation modes between experimental and FE simulation results. It can be seen that both the curve trend and amplitude, as well as the deformation modes, are approximately consistent, indicating that the material behaviour of rivet can be accurately simulated.

### 2.3. CFRP Plate

The upper CFRP plate for CFRP/Al SPR joint forming is made of T300 unidirectional fiber fabric and YG-04S epoxy resin. The plate has a layup of [0°]_6_ and a total thickness of 1.2 mm with each layer a thickness of 0.2 mm, which was produced by an CFRP product manufacturer from Harbin province, China. The mechanical properties were referenced from the parameters listed in the literature [20], as detailed in Table 1.

#### 2.3.1. Constitutive Model

The accuracy of CFRP material model directly affects the simulation results of CFRP/Al SPR joint forming. Unidirectional CFRP is generally considered elastic or-thogonal material, whose elastic constitutive relationship [18] can be expressed as:

(1) $$\sigma =\left[\begin{array}{l} \sigma_{11}\\ \sigma_{22}\\ \sigma_{33}\\ \sigma_{12}\\ \sigma_{23}\\ \sigma_{13} \end{array}\right]=C\cdot \varepsilon =\left[\begin{array}{cccccc} C_{11} & C_{12} & C_{13} & & & \\ C_{12} & C_{22} & C_{23} & & & \\ C_{13} & C_{23} & C_{33} & & & \\ & & & C_{44} & & \\ & & & & C_{55} & \\ & & & & & C_{66} \end{array}\right]\left[\begin{array}{l} \varepsilon_{11}\\ \varepsilon_{22}\\ \varepsilon_{33}\\ \varepsilon_{12}\\ \varepsilon_{23}\\ \varepsilon_{13} \end{array}\right]$$

where σ, ε and C denote the stress matrix, strain matrix and stiffness matrix, respectively. 1, 2, and 3 respectively represent the fiber direction (X), in-plane transverse direction (Y), and out-of-plane transverse direction (Z).

To accurately depict the damage evolution of CFRP materials, this study adopted a three-dimensional elastic damage constitutive model [18] as illustrated in Equation (2):

(2) $$\left[\begin{array}{l} \sigma_{11}\\ \sigma_{22}\\ \sigma_{33}\\ \sigma_{12}\\ \sigma_{23}\\ \sigma_{13} \end{array}\right]=\left[\begin{array}{cccccc} dC_{11} & dC_{12} & dC_{13} & & & \\ dC_{12} & dC_{22} & dC_{23} & & & \\ dC_{13} & dC_{23} & dC_{33} & & & \\ & & & dC_{44} & & \\ & & & & dC_{55} & \\ & & & & & dC_{66} \end{array}\right]\left[\begin{array}{l} \varepsilon_{11}\\ \varepsilon_{22}\\ \varepsilon_{33}\\ \varepsilon_{12}\\ \varepsilon_{23}\\ \varepsilon_{13} \end{array}\right]$$

where in-between matrix is the stiffness matrix considering the damage factor *d*. 

In the damage failure simulation, the CFRP is usually regarded as transverse isotropic material, whose mechanical behaviour is defined through the user subroutine (VUMAT) in Abaqus.

#### 2.3.2. Damage Initiation Criterion and Evolution

During the process of rivet leg piercing through the CFRP layers, various damage and failure modes could be often observed, including longitudinal tensile failure, longitudi-nal compression failure, transverse tensile failure, and transverse compression failure. To monitor the damage and failure behaviors of CFRP layers, the 3D Hashin damage initiation criterion based on strain was adopted in this study. Table 2 demonstrates the expressions for damage initiation, and when the material reaches the damage initiation, it enters to the damage evolution stage, and the corresponding damage evolution criteria are shown in Table 3, where the current equivalent plastic strains [18] can be expressed by Equation (3).

(3) $$\varepsilon_{i}^{f}=\frac{2G_{ic}}{f_{i}L_{k}},i=1t,1c,2t,2c$$

where $G_{ic}$ is the strain energy release rate and $L_{k}$ is the characteristic length of the element.

#### 2.3.3. Interlaminar Damage Model

To simulate the interlaminar delamination caused by the failure between layers in CFRP, this study employed the cohesive behavior command to define the interlayer contact relationship. Cohesive behavior simplifies the complex fracture problem using a macroscopic traction–separation criterion, whose constitutive relationship [18] could be presented as follows:

(4) $$\left(\begin{array}{c} t_{x}\\ t_{y}\\ t_{z} \end{array}\right)=\left[\begin{array}{ccc} E_{x} & & \\ & E_{y} & \\ & & E_{z} \end{array}\right]\left(\begin{array}{c} \varepsilon_{x}\\ \varepsilon_{y}\\ \varepsilon_{z} \end{array}\right)=\left[\begin{array}{ccc} K_{x} & & \\ & K_{y} & \\ & & K_{z} \end{array}\right]\left(\begin{array}{c} \delta_{x}\\ \delta_{y}\\ \delta_{z} \end{array}\right)$$

where t represents the stress components, E represents the elasticity modulus components, ε represents the strain components, K represents the stiffness parameter, δ represents the displacement components, and x, y, z represent the normal and two shear directions.

In this study, the quadratic stress failure criterion was adopted as the damage initiation criterion, which was expressed as:

(5) $$d_{c}={\left(\frac{t_{x}}{{t_{x}}^{\max}}\right)}^{2}+{\left(\frac{t_{y}}{{t_{y}}^{\max}}\right)}^{2}+{\left(\frac{t_{z}}{{t_{z}}^{\max}}\right)}^{2}\left\{\begin{array}{l} d_{c}<1,\;undamage\;\;\;\\ d_{c}\geq 1,\;\;\;\;\;damage \end{array}\right.\;\;\;$$

where ${t_{x}}^{\max}$, ${t_{y}}^{\max}$, ${t_{z}}^{\max}$ represent the maximum stress in each direction.

Upon reaching the damage initiation criterion, the damage evolution phase begins. This study employed the energy-based B-K criterion for damage evolution, as illustrated in Equation (6):

(6) $$G^{C}=G_{x}^{C}+\left(G_{y}^{C}-G_{x}^{C}\right){\left(\frac{G_{y}+G_{z}}{G_{x}+G_{y}+G_{z}}\right)}^{\eta }$$

where $G^{C}$ represents the equivalent fracture energy, $G_{x}^{C}$ is the mode I interlaminar fracture toughness, $G_{y}^{C}$ is the mode II interlaminar fracture toughness, $G_{x}$, $G_{y}$, $G_{z}$ represent the fracture energy in each direction, and η represents the material exponential constants. 

The interlaminar delamination damage parameters were referenced from the settings described in the literature [21], with the relevant parameters detailed in Table 4.

## 3. FE Modeling and Verification

### 3.1. Geometry Configuration

**Figure 7:**
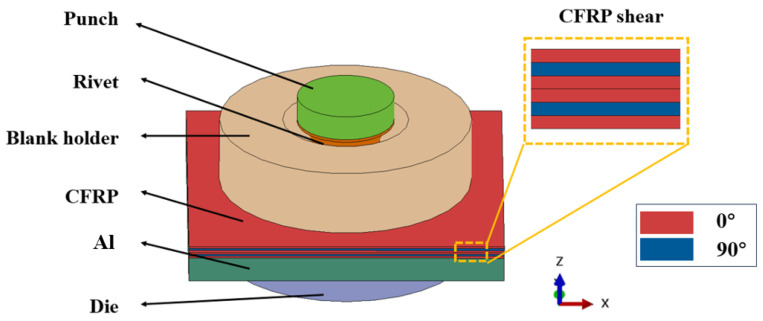
Geometric configuration of single lap CFRP/Al SPR joint before forming.

**Figure 8:**
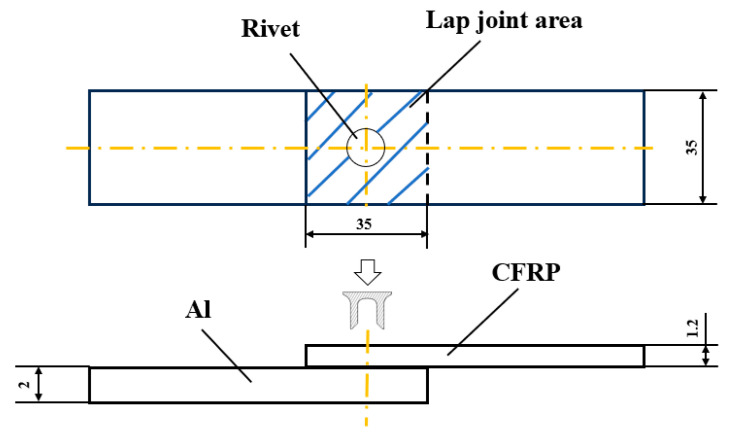
Geometric dimensions of single lap CFRP/Al SPR joint before forming.

**Figure 9:**
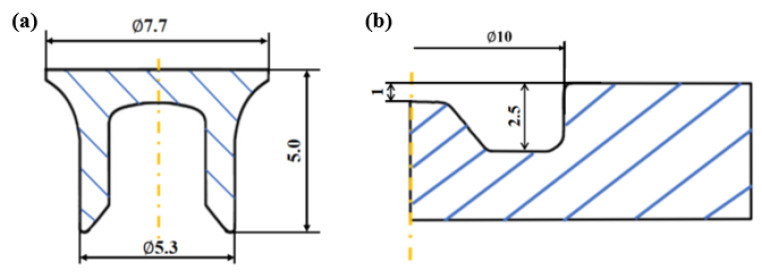
Geometric dimensions of (**a**) rivet and (**b**) die.

This study investigated the forming quality of SPR joint between CFRP and Al. Figure 7 demonstrates the geometric configuration diagram of a single lap SPR joint before forming. A pair of upper CFRP plate and lower Al plate was fixed in the middle by an upper blank holder and a lower die, and a semi-hollow self-piercing rivet was compressed by an upper punch to pierce through the upper CFRP plate and then formed self-locking through plastic deformation in the lower Al plate. The CFRP plate consists of six layers arranged in a [0°/90°/0°]_S_ sequence. Figure 8 and Figure 9 demonstrate the geometric dimensions of the single lap CFRP/Al SPR joint, the rivet and die. The length and width of the lap joint area were both 35 mm, and the thicknesses of CFRP plate and aluminium alloy plate were 1.2 mm and 2 mm, respectively.

### 3.2. FE Modeling

**Figure 10:**
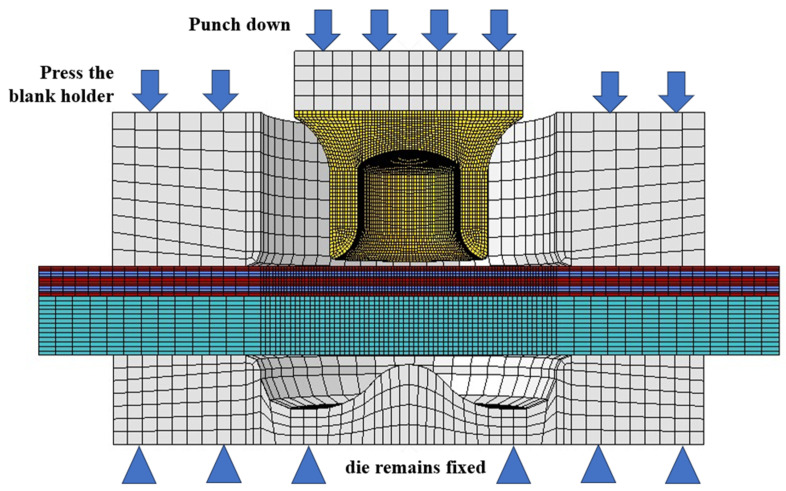
Self-punching riveting forming diagram.

The FE model of the single lap SPR joint forming was constructed by Abaqus, as shown in Figure 10. To enhance computational efficiency, the FE modelling was confined to the lap joint area, namely the dimensions of the upper CFRP plate and lower Al plate in the model were 35 mm × 35 mm × 1.2 mm and 35 mm × 35 mm × 2 mm, respectively. The die was fixed with all degree of freedom constrained, the blank holder was subjected to a downward force of 4500 N along the Z-axis with all other degrees of freedom constrained to prevent any lateral movement, and the punch was controlled to move downward using a smooth amplitude curve.

The CFRP plate, Al plate, self-piercing rivet, punch and die were all discretized using 8-node hexahedral linear reduced integration elements (C3D8R). To balance simulation accuracy and computational cost, a multi-scale grid partitioning strategy was adopted. Specifically, the mesh was refined at the center of the CFRP plate and the Al plate to improve the accuracy of the simulation results. The central regions of the CFRP and Al plates were meshed with element sizes of 0.1 × 0.1 × 0.1 mm and 0.15 × 0.15 × 0.15 mm, respectively, covering volumes of 10 mm × 10 mm × 1.2 mm for the CFRP plate and 10 mm × 10 mm × 2 mm for the Al plate. The rivet was meshed with element size of 0.12 mm × 0.12 mm × 0.12 mm. The total number of elements for the rivet, the CFRP plate and the Al plate sum up to 402,970. This meshing strategy ensured a high simulation accuracy in the critical forming areas, providing an accurate representation of the mechanical behaviors of connection materials during the riveting process.

The punch, blank holder, and die were modeled as rigid bodies since they do not deform during the riveting process. Additionally, for interactions between rigid and non-rigid bodies, the contact pairs were defined as surface-to-surface, with the rigid body set as the master surface to ensure accurate contact behaviors, while general contact was applied for other contact pairs. To prevent interpenetration between the layers of the CFRP plate, a general self-contact was implemented. Cohesive behavior contact interfaces were defined between each layer of the CFRP plate to accurately simulate the interlayer behavior. The friction coefficient between the rivet and the CFRP plate was set to 0, while the friction coefficients between all other components were set to 0.3 [12].

### 3.3. Verification of FE Models 

**Figure 11:**
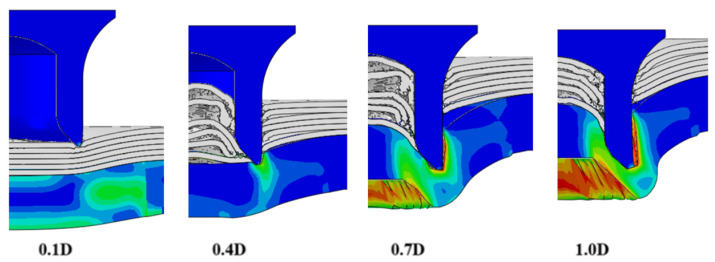
Deformation modes at joint’s central section under different riveting displacements.

**Figure 12:**
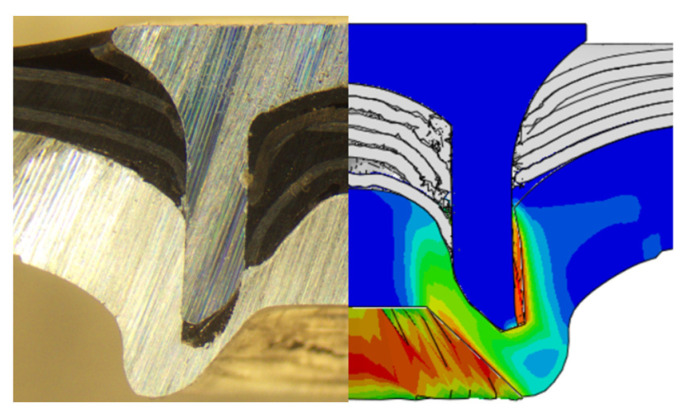
Comparison of the joint’s sectional deformation between simulation and experiment.

**Figure 13:**
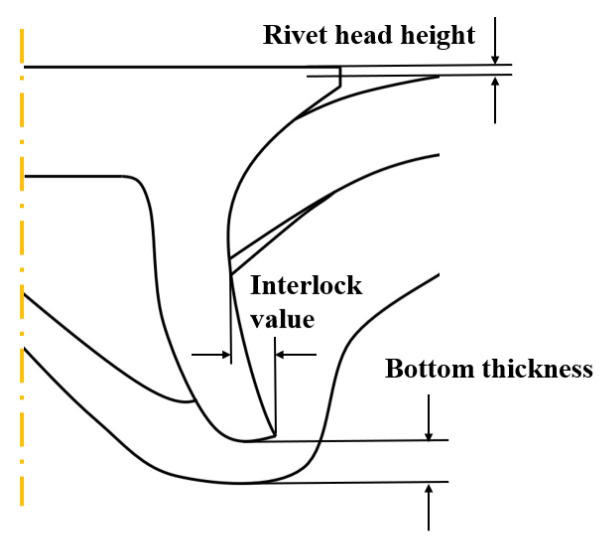
Forming quality indicators at joint’s central section.

To validate the accuracy of the FE model of CFRP/Al SPR joint forming, the FE numerical simulation of SPR joint forming was first conducted, and the joint’s deformation modes at central section under different riveting displacements (0.1D, 0.4D, 0.7D and 1.0D) during forming process are shown in Figure 11, where D demotes the total compression stroke of punch. In the meantime, the CFRP and aluminium alloy plates were manufactured and the CFRP/Al SPR joint forming test was also conducted using a self-piercing riveting testing machine. The SPR joint was then cut from the middle plane using a cutting machine to demonstrate the sectional deformation, which was compared with that of simulation result, as shown in Figure 12. It can be seen that, the sectional deformation mode of simulation is basically consistent with that of experiment. In addition, the values of three commonly employed forming quality indicators of SPR joint, i.e., bottom thickness (BT) [22,23], interlock value (IV) [24,25], and rivet head height (RHH), which are three sectional parameters of joint as shown in Figure 13, were extracted for further comparison, as listed in Table 5. According to the outcomes, the relative errors between simulation and experiment for BT, IV and RHH are −5.31%, −5.00%, and 0%, respectively, absolute values of which are all less than 6%, indicating that the established FE model of CFRP/Al SPR joint forming has good accuracy and is suitable for further parameter study and optimization of forming quality.

## 4. Parameter Study

**Figure 14:**
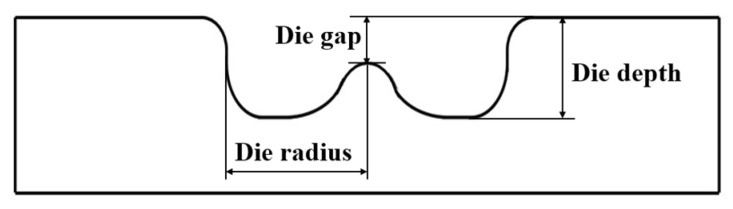
Die structure diagram.

To thoroughly understand the forming characteristics of CFRP/Al SPR joint, this study systematically investigated the effects of key forming process parameters, i.e., die depth ($D_{d}$), die gap ($D_{g}$), die radius ($D_{r}$), and rivet length ($R_{L}$), on the joint’s forming quality. The die structure diagram and the above three parameters ($D_{d}$, $D_{g}$ and $D_{r}$) are shown in Figure 14. According to previous research, the RHH has a minimal effect on the joint’s connection strength [26], therefore the forming indicators considered in this study were limited to the BT and IV. Note that for SPR joint, both the BT [16] and IV [17] are larger-the-better to increase its connection performance, hence the larger the BT or IV, the better the forming quality. In addition, note that the effect of rivet length ($R_{L}$) on joint’s forming quality indicators are stacked in one paragraph with other die parameters, hence it is no longer listed and discussed separately.

### 4.1. The Effect of Die Depth ($D_{d}$)

**Figure 15:**
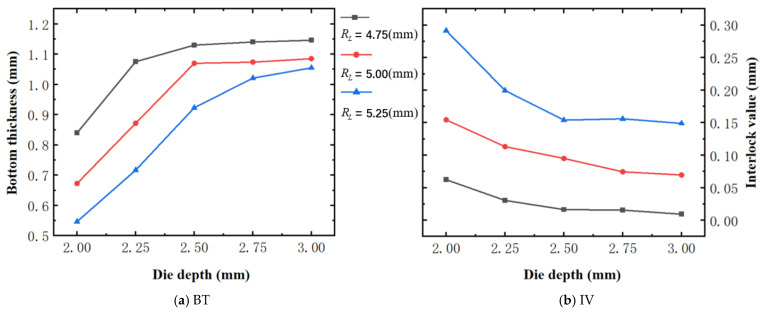
Forming quality indicators of joint with different $D_{d}$ and $R_{L}$.

**Figure 16:**
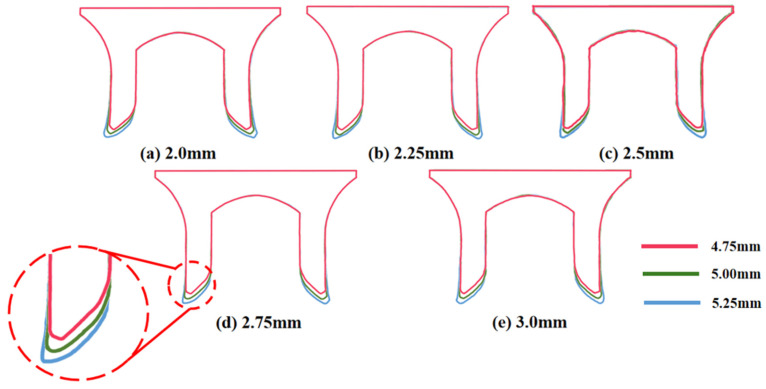
Overlay image of rivet’s sectional deformation contours with different $D_{d}$ and $R_{L}$.

Figure 15 shows the variation of BT and IV of the SPR joint with different $D_{d}$ (2.0 mm, 2.25 mm, 2.5 mm, 2.75 mm, and 3.0 mm) and $R_{L}$ (4.75 mm, 5.00 mm, and 5.25 mm). In Figure 15a, the BT of joint with any $R_{L}$ all increases with the increase of $D_{d}$. For joints with $R_{L}$ of 4.75 mm and 5.00 mm, their BT firstly increase rapidly and then increase slowly. In contrast, for joint with $R_{L}$ of 5.25 mm, its BT follows a more linear increasing trend across the entire range of $D_{d}$. In Figure 15b, the IV of joint with any $R_{L}$ approximately all decreases with the increase of $D_{d}$. For joint with $R_{L}$ of 5.25 mm, its IV firstly decreases rapidly and then decreases slowly. For joints with smaller $R_{L}$ (4.75 mm and 5.00 mm), the decreases in IV are more gradual throughout the range of $D_{d}$. In addition, it can be seen that for a joint with any $D_{d}$, its BT decreases with the increase of $R_{L}$, while its IV increases with the increase of $R_{L}$, which can be more intuitively explained by the overlay image of rivet’s deformation contours with different $D_{d}$ and $R_{L}$ are shown in Figure 16, where the change of deformation mode of the rivet head can well reflects the above conclusion. Furthermore, for a more intuitive presentation, the corresponding sectional deformation modes of joints with different $D_{d}$ and $R_{L}$ are also compared and listed in Table 6, where some difference in deformation modes between different joints can also be discovered.

According to above results, it can be seen that with the independent increase of either $D_{d}$ or $R_{L}$, the two forming quality indicators (BT, IV) demonstrate an opposite trend of change, indicating the two forming indicator are competitive or even contradictory, and the effect of either $D_{d}$ or $R_{L}$ on the joint’s overall forming quality is uncertain.

### 4.2. The Effect of Die Gap ($D_{g}$)

**Figure 17:**
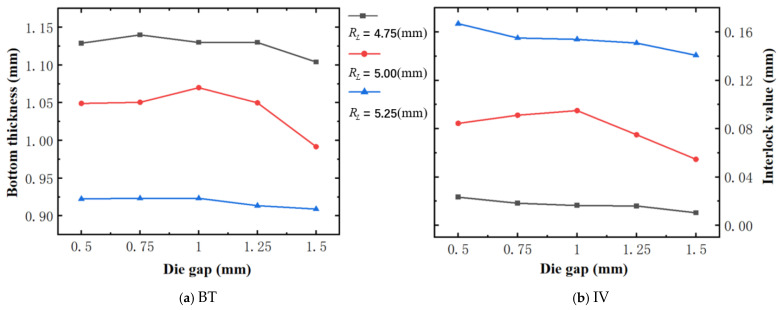
Forming indicators of joint with different $D_{g}$ and $R_{L}$.

Figure 17 illustrates the variation of BT and IV of the SPR joint with different $D_{g}$ (0.5 mm to 1.5 mm) and $R_{L}$ (4.75 mm, 5.00 mm, and 5.25 mm). As shown in Figure 17a, the trends of BT with the increase of $D_{g}$ vary with different $R_{L}$. Specifically, for joint with $R_{L}$ of 4.75 mm, its BT firstly increases and then decreases with the increase of $D_{g}$. In contrast, for joints with $R_{L}$ of 5.00 mm and 5.25 mm, their BT both firstly increase and then decrease with the increase of $D_{g}$. As shown in Figure 17b, the trends of IV with the increase of $D_{g}$ also vary with different $R_{L}$. The IV of the joints with rivet length of 4.75 mm and 5.25 mm both decreases slowly with the increase of $D_{g}$. In contrast, the IV of the joint with $R_{L}$ of 5.00 mm firstly increases and then decreases, with the increase of $D_{g}$. Also, it can be seen that for a joint with any $D_{g}$, its BT decreases with the increase of $R_{L}$, while its IV increases with the increase of $R_{L}$, just as discussed above. Likewise, for a more intuitive presentation, the corresponding sectional deformation modes of joints with different $D_{g}$ and $R_{L}$ are also compared and listed in Table 7, where some difference in deformation modes between different joints can also be discovered.

According to above results, for joint with $R_{L}$ of 5.00 mm, the joint’s forming quality firstly improves and then deteriorates with the increase of $D_{g}$. Apart from this, the effects of $D_{g}$ on the overall forming quality of joints with other $R_{L}$ (4.75 mm and 5.25 mm) are uncertain. Of course, for the joint with any $D_{g}$, the effect of $R_{L}$ on the joint’s overall forming quality is uncertain. In addition, the competition and contradiction are once again verified.

### 4.3. The Effect of Die Radius ($D_{r}$)

**Figure 18:**
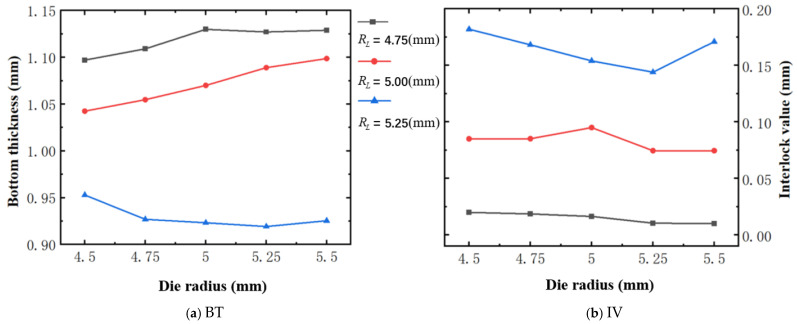
Forming indicators of joint with different $D_{r}$ and $R_{L}$.

Figure 18 illustrates the variation of BT and IV of the SPR joint with different $D_{r}$ (4.5 mm to 5.5 mm) and $R_{L}$ (4.75 mm, 5.00 mm, and 5.25 mm). As shown in Figure 18a, the trends of BT with the increase of $D_{r}$ vary with different $R_{L}$. Specifically, for joint with $R_{L}$ of 4.75 mm, its BT firstly increases rapidly and then keeps basically constant with the increase of $D_{r}$. In contrast, for joint with $R_{L}$ of 5.25 mm, the BT firstly decreases rapidly and then increases slowly with the increase of $D_{r}$. As for joint with $R_{L}$ of 5.00 mm, its BT monotonically increases with the increase of $D_{r}$. As shown in Figure 18b, the trends of IV with the increase of $D_{r}$ vary with different $R_{L}$. For joint with $R_{L}$ of 4.75 mm, its IV slightly decrease with the increase of $D_{r}$. For joint with $R_{L}$ of 5.00 mm, its IV firstly increases slowly and then decreases slowly with the increase of $D_{r}$. As for joint with $R_{L}$ of 5.25 mm, its IV firstly decreases sharply and then increases sharply with the increase of $D_{r}$. Also, it can be seen that for a joint with any $D_{r}$, its BT decreases with the increase of $R_{L}$, while its IV increases with the increase of $R_{L}$, which is confirmed again. Likewise, for a more intuitive presentation, the corresponding sectional deformation modes of joints with different $D_{r}$ and $R_{L}$ are also compared and listed in Table 8, where some difference in deformation modes between different joints can also be discovered.

## 5. Multi-Objective Optimization of Joint’s Forming Quality

For SPR joint, optimizing the forming process parameters to improve its forming quality can effectively enhance its joint connection performance, which thus is worthy of further research. As aforementioned, both BT and IV are larger-the-better to improve the joint’s forming quality and thus were all taken as optimization objectives. However, as indicated by the previous analysis, different parameters have distinct effects on forming quality indicators BT and IV, which present a competitive or even contradictory relationship. Traditional single-objective optimization methods are unable to handle such kind of multi-objective optimization issue of achieving optimization of the two indicators simutaneously [27], necessitating the exploration of multi-objective optimization approach for the joint’s forming quality. Unlike the time-consuming and labor-intensive surrogate model-based optimization methods, this study proposes a hybrid Taguchi-TOPSIS approach to conduct the optimization. 

### 5.1. Taguchi Method Coupled with TOPSIS

The detailed process of the Taguchi method coupled with TOPSIS is described as follows:

Step 1: Taguchi experimental design

In this study, the above four forming process parameters, i.e., rivet length ($R_{L}$), die depth ($D_{d}$), die gap ($D_{g}$), and die radius ($D_{r}$) were taken as design factors, for each of which four distinct levels were set, as shown in Table 9. To systematically investigate the effects of these factors, an $L_{16}(4^{4})$ orthogonal array was employed. This array, designed according to Taguchi’s method, efficiently reduces the number of required experiments while ensuring a comprehensive exploration of the factor space. The orthogonal array design allows for the examination of the main effects with a minimal number of experimental runs, thus saving time and resources.

Table 10 outlines the specific configuration of the Taguchi experiments, detailing the combination of factor levels for each experimental sample, and the corresponding experimental results for the responses: BT and IV, which were all taken as optimization objectives. These results will form the basis for the multi-objective optimization process in the subsequent steps. 

Step 2: Signal-to-noise ratio (*SNR*)

The Taguchi method utilizes the signal-to-noise ratio (*SNR*) to evaluate the performance of responses, effectively accounting for the variability encountered across a set of experimental runs. Different *SNR* calculation methods were employed based on the nature of the response characteristics. Specifically:

For responses exhibiting the “larger-the-better” (LTB) characteristic, their *SNR*s could be calculated as [28]:

(7) $$SNR_{\mathrm{LTB}}=-10\log_{10}(\frac{1}{t}\sum_{i=1}^{t}\frac{1}{{x_{i}}^{2}})$$

For responses exhibiting the “smaller-the-better” (STB) characteristic, their *SNR*s could be calculated as:

(8) $$SNR_{\mathrm{STB}}=-10\log_{10}(\frac{1}{t}\sum_{i=1}^{t}{x_{i}}^{2})$$

where $x_{i}$ is the experimental result of response x at the ith measurement, t is the total number of measurements per experiment.

In this study, for the multi-objective optimization of the CFRP/Al SPR joint forming, the evaluated responses, i.e., BT and IV, both exhibit “larger-the-better” (LTB) characteristics. Thus, the *SNR* values for BT and IV, denoted as *SNR*1 and *SNR*2, respectively, were calculated using the LTB formula (Equation (7)) and listed as shown in Table 10.

Step 3: Technique for Order Preference by Similarity to Ideal Solution (TOPSIS)

Normally, the Taguchi method could evaluate and compare the influence of each factor on the response, and then further determine the optimal combination of factor levels for a single-objective optimization [29].However, as aforementioned earlier, the traditional Taguchi method was initially introduced to optimize a single response at a time, which is not suitable to optimize multiple responses simultaneously. Hence, TOPSIS was incorporated to Taguchi method to transform the multiple responses to a single relative closeness coefficient (RCC) that is to be maximized. The TOPSIS method is a useful technique for solving multi-criteria decision-making (MCDM) problems by ranking the possible solutions through the measurement of Euclidean distance to explore the optimal alternative [30,31]. The detailed steps are as follows:

Extract *SNR*1 and *SNR*2 data to construct a decision making matrix, which can be expressed as:

(9) $$Y=\left(\begin{array}{cccc} y_{11} & y_{12} & & y_{1n}\\ y_{21} & y_{22} & & y_{2n}\\ & & \ddots & \\ y_{m1} & y_{m2} & & y_{mn} \end{array}\right)$$

where $y_{ij}(i=1,2,\cdots ,m;j=1,2,\cdots ,n)$ denote the *SNR* of jth response for ith experiment, m represents the number of alternatives and n represents the number of objectives.

To ensure comparability of the *SNR* values for BT (*SNR*1) and IV (*SNR*2), the defined decision making matrix is normalized to the non-scaled matrix using the following:

(10) $$z_{ij}=y_{ij}/\sqrt{\sum_{i=1}^{m}y_{ij}^{2}}(i=1,2,\ldots ,m;\;j=1,2,\ldots ,n)$$

The normalized matrix is denoted as Z and can be expressed as:

(11) $$Z=\left(\begin{array}{cccc} z_{11} & z_{12} & & z_{1n}\\ z_{21} & z_{22} & & z_{2n}\\ & & \ddots & \\ z_{m1} & z_{m2} & & z_{mn} \end{array}\right)$$

Generally, the relative importance of each responses may be different. The corresponding weights of the objectives can be calculated using entropy method, which is a measure of uncertainty in the information formulated using probability theory. The entropy value $e_{j}$ can be determined as:

(12) $$e_{j}=-\frac{\sum_{i=1}^{m}p_{ij}\ln p_{ij}}{\ln(m)}$$

where $p_{ij}$ is the projection value of $z_{ij}$, and it can be expressed as follows:

(13) $$p_{ij=}\frac{z_{ij}}{\sum_{i=1}^{m}z_{ij}}$$

The weight for each responses can be expressed as:

(14) $$w_{j}=\frac{d_{j}}{\sum_{j=1}^{n}d_{j}}$$

where $d_{j}=1-e_{j}$ is the deviation degree of the jth optimization objective. Generally speaking, a higher degree of deviation indicates that it provides more information, and thus the objective associated with it will be given a higher weight. 

The weight normalized value $u_{ij}$ can be calculated as:

(15) $$u_{ij}=w_{j}z_{ij}\;\;\sum_{j=1}^{n}w_{j}=1$$

where $w_{j}$ is the weight of the jth objective.

Now, the positive ideal solution (PIS) and negative ideal solution (NIS) are determined for calculating the distance of an alternative from the best and worst altermatives. The PIS ($U^{+}$) and NIS ($U^{-}$) can be determined as follows:

(16) $$\left\{\begin{array}{c} U^{+}=\left\{u_{1}^{+},u_{2}^{+},\cdots ,u_{n}^{+}\right\}\\ U^{-}=\left\{u_{1}^{-},u_{2}^{-},\cdots ,u_{n}^{-}\right\} \end{array}\right.$$

If the objective function is to be maximized, which means the characteristic is larger-the-better, the PIS and NIS are respectively determined by:

(17) $$\left\{\begin{array}{c} u_{j}^{+}=\max_{i}\left\{u_{ij},j=1,2,\cdots ,n\right\}\\ u_{j}^{-}=\min_{i}\left\{u_{ij},j=1,2,\cdots ,n\right\} \end{array}\right.$$

The separation of each alternative from PIS and NIS can be determined by Euclidean distance, which are defined by Equation (18).

(18) $$\left\{\begin{array}{c} D_{i}^{+}=\sqrt{\sum_{j=1}^{n}{\left(u_{ij}-u_{j}^{+}\right)}^{2}}\\ D_{i}^{-}=\sqrt{\sum_{j=1}^{n}{\left(u_{ij}-u_{j}^{-}\right)}^{2}} \end{array}\right.$$

where $D_{i}^{+}$ represents the distance between the ith alternative and the PIS, $D_{i}^{-}$ denotes the distance between the ith alternative and the NIS.

Finally, the relative closeness coefficient (RCC) between each alternative and the PIS is calculated as follows:

(19) $$C_{i}=\frac{D_{i}^{-}}{D_{i}^{+}+D_{i}^{-}}$$

where $C_{i}$ is the RCC of ith alternative.

Normally, the larger the $C_{i}$, the closer it is to the PIS, indicating the alternative is relatively better. In Taguchi’s experimental samples, the alternative with the largest value of $C_{i}$ is the best choice.

Step 4: Main effect analysis

To further understand the influence of each design variable on the forming quality indicators of the CFRP/Al SPR joint, main effect analysis was conducted. With the help of TOPSIS, the multiple optimization objectives of the forming quality indicator of CFRP/Al SPR joint were transformed into a single evaluation index. 

This allows the Taguchi analysis to explore the optimal combination of factor levels. First, the average RCC response values for each level of the design variables (rivet length, die depth, die gap, and die radius) were calculated. The main effect of each factor on the RCC was then determined by calculating the difference between the maximum and minimum average RCC values for each factor. This difference indicates the primary influence of each factor on the RCC, which could be described as follows:

(20) $${\bar{f}}_{p}=(\frac{1}{W})\sum_{q=1}^{W}\Gamma_{pq}$$

(21) $$\Delta f=\max({\bar{f}}_{1},{\bar{f}}_{2}\cdot \cdot \cdot ,{\bar{f}}_{U})-\min({\bar{f}}_{1},{\bar{f}}_{2}\cdot \cdot \cdot ,{\bar{f}}_{U})$$

where ${\bar{f}}_{p}$ represents the average RCC value for factor f at the pth level, $\Gamma_{pq}$ represents the RCC of the qth experiment with factor f at the pth level, W represents the total number of experiments with factor f at the pth level, U represents the total number of levels of factor f, and Δf represents the main effect value of factor f.

In practice, the effect of the average response values (${\bar{f}}_{p}$) for each factor level and the main effect values (Δf) of each factor on the response can be obtained from the factor response table and main effect plot, respectively. These plots and tables provide insights into the main effects of the factors on the response. Specifically, the main effect plot helps to identify the factor levels that most significantly improve the overall response. A key outcome of this analysis is the identification of the optimal combination of factor levels for the best response. Generally, a lower main effect value indicates a smaller impact of that factor on the response, and vice versa.

### 5.2. Results and Discussion

Table 10 presents the signal-to-noise ratios (*SNR*1 for BT and *SNR*2 for IV) calculated for each result based on Equation (7). The *SNR* response tables for BT and IV are shown in Table 11 and Table 12, respectively. For a given factor, the mean *SNR* at a specific level represents the overall average *SNR* for all experiments at that level, while the maximum difference (Delta) between levels indicates the primary effect of that factor on the *SNR*. Generally, factors with higher Delta values exert a relatively greater influence on the response.

**Figure 19:**
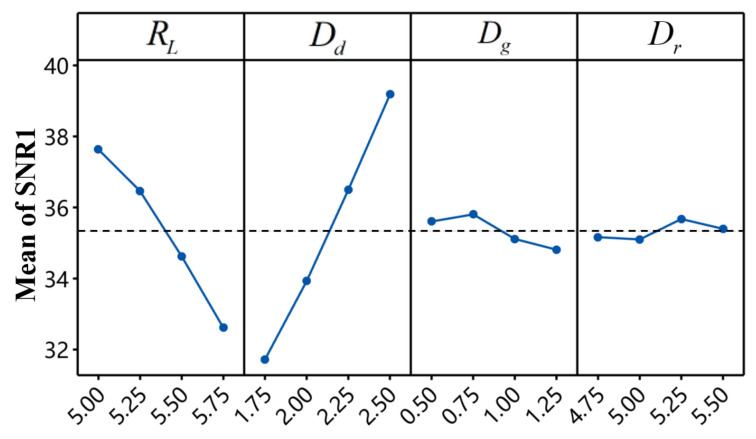
Main effects plot for *SNR*1.

**Figure 20:**
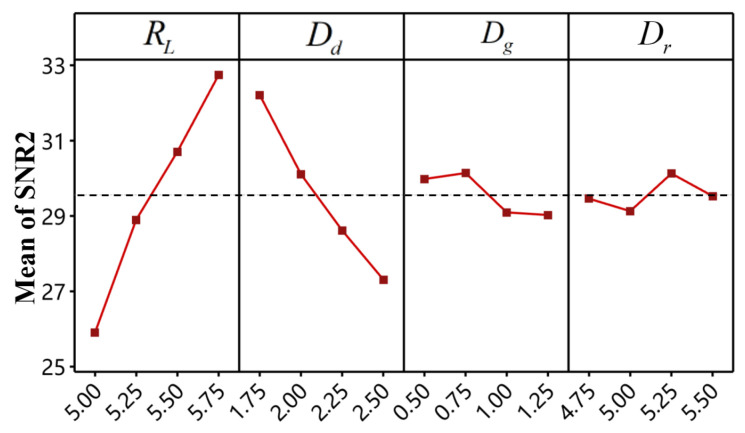
Main effects plot for *SNR*2.

As shown in Table 11, die depth is the most significant factor affecting *SNR*1, followed by rivet length, die gap, and die radius. In contrast, Table 12 indicates that rivet length has the greatest impact on *SNR*2, followed by die depth, die gap, and die radius. The main effect plots for *SNR*1 and *SNR*2 are illustrated in Figure 19 and Figure 20, respectively, where steeper slopes signify a greater influence of that factor on the response. These plots also reveal the optimal combination of factor levels for each response. Specifically, for maximizing BT, the optimal factor levels are $R_{L}1-D_{d}4-D_{g}2-D_{r}3$, as highlighted in bold in Table 11. Similarly, for maximizing IV, the optimal combination is $R_{L}4-D_{d}1-D_{g}2-D_{r}3$, as highlighted in bold in Table 12.

In summary, the optimal factor level combinations for maximizing *SNR*1 (BT) and *SNR*2 (IV) are different. Some factors exhibit completely opposite trends, such as rivet length and die depth. Consequently, the traditional Taguchi method of optimizing a single response at a time is not enough for the multi-objective optimization problem of improving the BT and IV of the CFRP/Al SPR joint at the same time. Therefore, in order to promote the multi-objective optimization of the forming characteristics of CFRP/Al SPR joint, this paper adopted the TOPSIS to convert the signal-to-noise ratio of the two forming indicators (BT and IV) into a single response, that is, the relative closeness coefficient (RCC).

**Figure 21:**
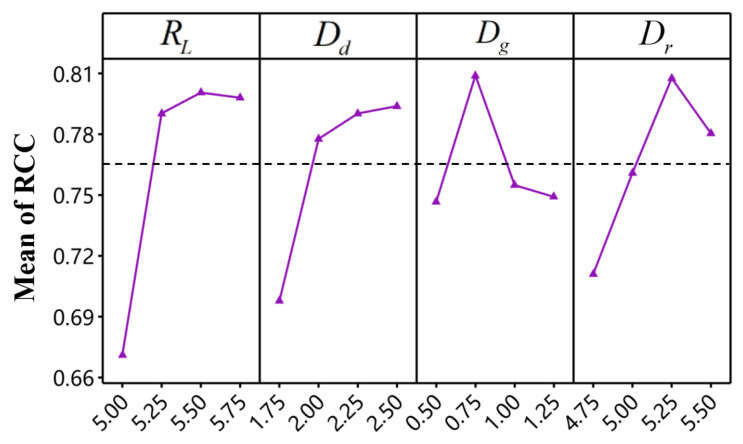
Main effect plots of factors on RCC.

Table 13 lists the normalized values ($z_{ij}$) calculated based on Equation (9), the weighted values ($u_{ij}$) obtained by weighting the normalized values based on Equation (15), and the relative closeness coefficient (RCC) based on Equation (18). Similarly, the response table for RCC (LTB) can be derived from Table 13, as shown in Table 14. The factor that has the most significant impact on RCC is the rivet length, followed by die radius, die depth, and die gap. Additionally, Figure 21 illustrate the main effect plot. From Figure 21, it is evident that the optimal factor level combination for maximizing RCC is $R_{L}3-D_{d}4-D_{g}2-D_{r}3$ (as indicated in bold in Table 14).

### 5.3. Optimization Results Validation

**Figure 22:**
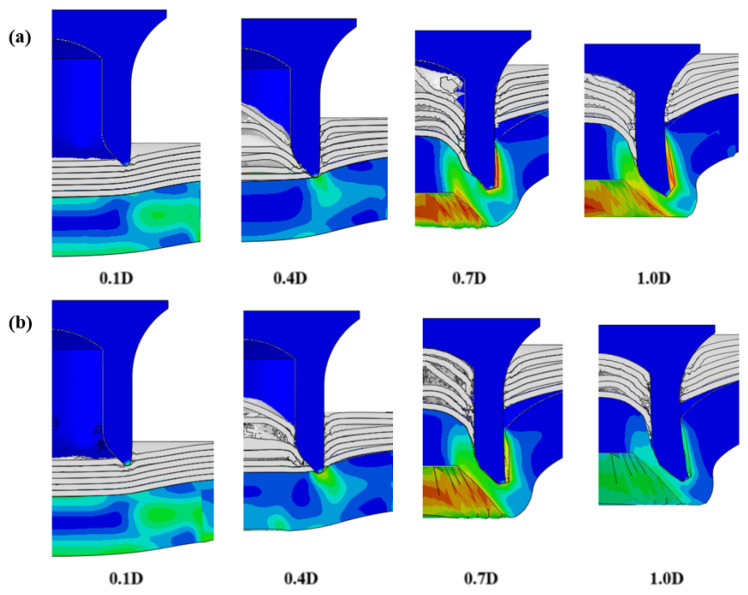
Comparison of sectional deformation modes under different riveting displacements: (**a**) Initial design; (**b**) Optimized design.

**Figure 23:**
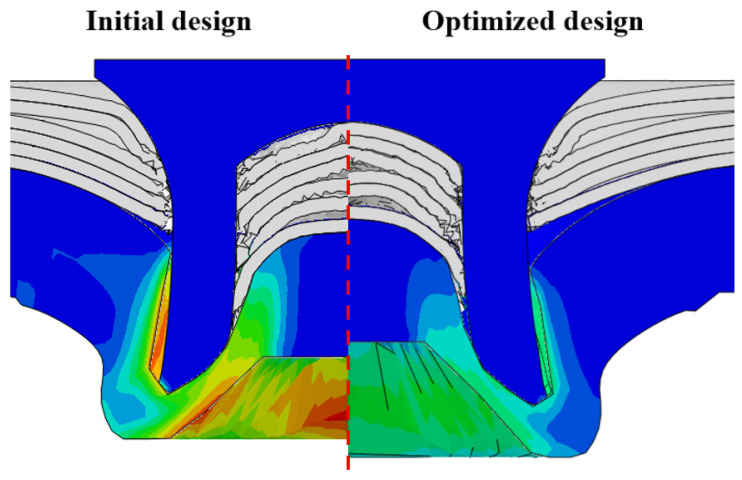
Comparison of joint’s final sectional deformation model before and after optimization.

After determining the optimal factor level combination for CFRP/Al SPR joint, this section analysed the effectiveness of the optimized results. Specifically, the forming quality of the optimized design ($R_{L}3-D_{d}4-D_{g}2-D_{r}3$) and the initial design ($R_{L}2-D_{d}3-D_{g}3-D_{r}2$) were compared. The sectional deformation modes under different riveting displacements before and after optimization were compared in Figure 22, while the final sectional deformation modes before and after optimization were compared in Figure 23. Moreover, the joint’s forming quality indicators were compared in Table 15. It can be seen that compared with the initial design, the optimized design improved the BT and IV by 10.14% and 34.17%, respectively, which could also be reflected by the joint’s sectional deformation modes and rivet’s sectional deformation contours, indicating a good multi-objective optimization of the forming quality of the CFRP/Al SPR joint was achieved.

## 6. Conclusions

This study systematically investigated the effects of forming process parameters on the forming quality of CFRP/Al SPR joint, and then conducted a further multi-objective optimization of forming quality using a proposed hybrid Taguchi–TOPSIS method. The main research findings can be summarized as follows:

(1) The FE model of CFRP/Al SPR joint forming is built to monitor the actual joint’s forming process, and the relative errors between simulation and experiment for BT, IV and RHH are −5.31%, −5.00%, and 0%, respectively, absolute values of which are all less than 6%, indicating that the established FE model of CFRP/Al SPR joint forming has good accuracy and is suitable for further parameter study and optimization of forming quality.

(2) According to the parameter study, the BT increases while IV decreases with the increase of die depth, while the BT decreases while IV increases with the increase of rivet length. As to die gap and die radius, their independent effects on BT or IV are uncertain and depend on the rivet length used. As to the joint’s overall forming quality, namely taking BT and IV into simultaneous account, the effects of above parameters on the joint’s forming quality is uncertain. 

(3) According to the main effect analysis of *SNR*1 and *SNR*2, the factor influence ranking for BT is die depth, rivet length, die gap, and die radius; while for IV, the ranking is rivet length, die depth, die gap, and die radius.

(4) A hybrid Taguchi-TOPSIS method is proposed to conduct the multi-objective optimization of forming quality of CFRP/Al joint. According to the optimization outcomes, the optimized design improves the BT and IV by 10.14% and 34.17%, respectively, compared with the initial design, indicating a good multi-objective optimization of the forming quality of the CFRP/Al SPR joint is achieved, which provides an effective new method for efficiently predicting and improving the forming quality of CFRP/Al joint.

## Figures and Tables

**Table 1:** Material properties of CFRP.

Material Constant	Value	Material Constant	Value
Young’s modulus (GPa)	$\begin{array}{l} E_{11}=144\\ E_{22}=E_{33}=7.6 \end{array}$	Tensile strength (MPa)	$\begin{array}{l} f_{1t}=2250\\ f_{2t}=f_{3t}=43 \end{array}$
Shear modulus (GPa)	$\begin{array}{l} G_{12}=G_{13}=3.85\\ G_{23}=2.85 \end{array}$	Compressive strength (MPa)	$\begin{array}{l} f_{1c}=906\\ f_{2c}=f_{3c}=156 \end{array}$
Poisson’s ratio	$\lambda_{12}=\lambda_{13}=\lambda_{23}=0.31$	Shear strength (MPa)	$\begin{array}{l} f_{12}=f_{13}=65.7\\ f_{23}=37.9 \end{array}$

**Table 2:** Initial damage criterion based on strain.

Failure Mode	Initial Damage Criterion
Fiber tensile damage ($\varepsilon_{11}\geq 0$)	$r_{1t}={\left(\varepsilon_{11}\cdot \frac{C_{11}}{f_{1t}}\right)}^{2}+{\left(\varepsilon_{12}\cdot \frac{2G_{12}}{f_{12}}\right)}^{2}+{\left(\varepsilon_{13}\cdot \frac{C_{13}}{f_{13}}\right)}^{2}$
Fiber compressive damage ($\varepsilon_{11}<0$)	$r_{1c}={\left(\varepsilon_{11}\cdot \frac{C_{11}}{f_{1c}}\right)}^{2}$
Matrix tensile damage ($\varepsilon_{22}+\varepsilon_{33}\geq 0$)	$r_{2t}={\left(\frac{\varepsilon_{22}+\varepsilon_{33}}{f_{2t}/C_{22}}\right)}^{2}+\frac{\varepsilon_{23}^{2}-\varepsilon_{22}\varepsilon_{33}}{\left(f_{23}/2G_{23}\right)}+{\left(2\varepsilon_{12}\frac{G_{12}}{f_{12}}\right)}^{2}+{\left(2\varepsilon_{13}\frac{G_{13}}{f_{13}}\right)}^{2}$
Matrix compressive damage ($\varepsilon_{22}+\varepsilon_{33}<0$)	$\begin{array}{cc} r_{2c} & ={\left(\varepsilon_{22}+\varepsilon_{33}\right)}^{2}\cdot \left(\frac{C_{22}}{f_{2c}}\cdot \frac{C_{33}}{f_{3c}}\right)+\left(\varepsilon_{22}+\varepsilon_{33}\right)\cdot \left(\frac{f_{2c}}{C_{22}}\cdot \frac{G_{12}}{f_{12}}-1\right)\left(\frac{C_{22}}{f_{2c}}\right)\\ & -2\left(\varepsilon_{22}\varepsilon_{33}\cdot \frac{G_{13}}{f_{23}}\right)+{\left(2\varepsilon_{12}\cdot \frac{G_{12}}{f_{12}}\right)}^{2}+{\left(2\varepsilon_{13}\cdot \frac{G_{13}}{f_{13}}\right)}^{2}+{\left(2\varepsilon_{23}\cdot \frac{G_{23}}{f_{23}}\right)}^{2} \end{array}$

where $f_{1t}$, $f_{1c}$ are the tensile strength and compressive strength in fiber direction (X), respectively; $f_{2t}$, $f_{2c}$ are the tensile strength and compressive strength in Y and Z directions; and $f_{12}$, $f_{23}$, $f_{13}$ are the shear strengths in the three directions.

**Table 3:** Damage evolution criterion based on strain.

Failure Mode	Damage Evolution Criterion
$\varepsilon_{11}\geq 0$	$d_{1t}=\varepsilon_{1t}^{f}\left(1-\frac{f_{1t}/C_{11}}{\varepsilon_{11}}\right)/\varepsilon_{1t}^{f}-f_{1t}/C_{11}$
$\varepsilon_{11}<0$	$d_{1c}=\varepsilon_{1c}^{f}\left(1-\frac{f_{1c}/C_{11}}{\varepsilon_{11}}\right)/\varepsilon_{1c}^{f}-f_{1c}/C_{11}$
$\varepsilon_{22}+\varepsilon_{33}\geq 0$	$d_{2t}=\varepsilon_{2t}^{f}\left(1-\frac{f_{2t}/C_{22}}{\left|\varepsilon_{22}\right|}\right)/\varepsilon_{2t}^{f}-f_{2t}/C_{22}$
$\varepsilon_{22}+\varepsilon_{33}<0$	$d_{2c}=\varepsilon_{2c}^{f}\left(1-\frac{f_{2c}/C_{22}}{\left|\varepsilon_{22}\right|}\right)/\varepsilon_{2c}^{f}-f_{2c}/C_{22}$

where $\varepsilon_{i}^{f}(i=1t,1c,2t,2c)$ represent the current equivalent plastic strains.

**Table 4:** Interlaminar delamination damage parameters of CFRP.

${t_{x}}^{max}$ (MPa)	${t_{y}}^{max}$ (MPa)	${t_{z}}^{max}$ (MPa)	$G_{x}^{C}$ (J/m^2^)	$G_{y}^{C}$ (J/m^2^)	$G_{z}^{C}$ (J/m^2^)
72	72	72	272	748	748

**Table 5:** Comparison of forming quality indicators between simulation and experiment.

Geometric Dimension	BT	IV	RHH
Experiment (mm)	1.13	0.1	0.36
Simulation (mm)	1.07	0.095	0.36
Relative error	−5.31%	−5.00%	0%

**Table 6:** Comparison of joint’s sectional deformation modes with different $R_{L}$ and $D_{d}$.

Rivet Length ($R_{L}$)/mm	Die Depth ($D_{d}$)/mm				
	2.00	2.25	2.50	2.75	3.0
**4.75**	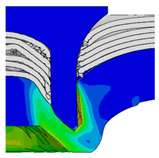	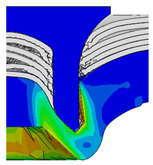	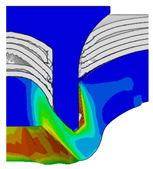	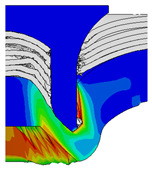	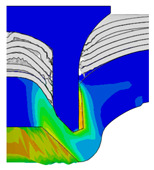
**5.00**	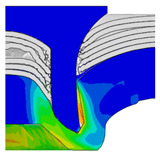	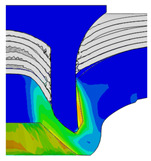	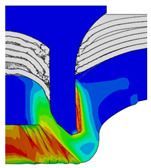	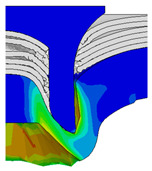	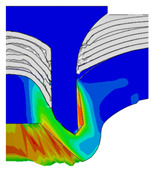
**5.25**	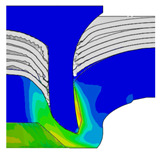	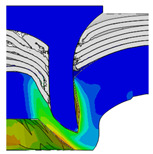	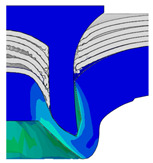	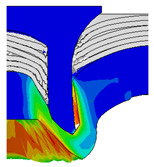	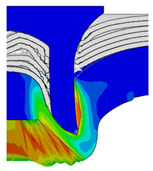

**Table 7:** Comparison of joint’s sectional deformation modes with different $D_{g}$ and $R_{L}$.

Rivet Length ($R_{L}$)/mm	Die Gap ($D_{g}$)/mm				
	0.50	0.75	1.00	1.25	1.50
**4.75**	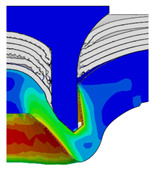	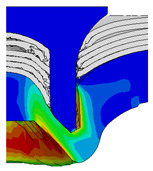	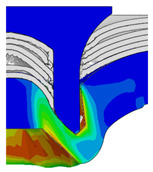	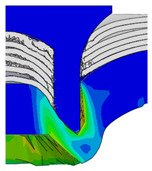	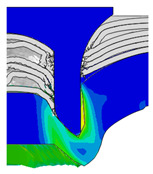
**5.00**	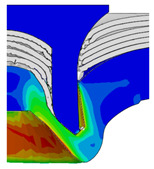	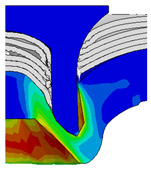	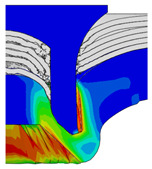	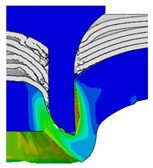	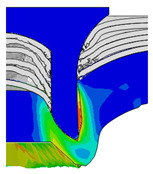
**5.25**	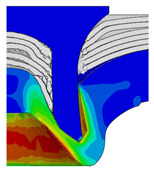	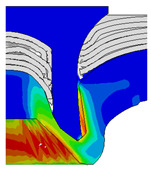	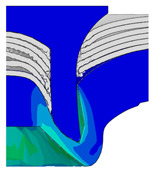	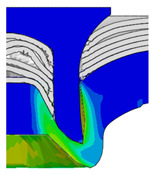	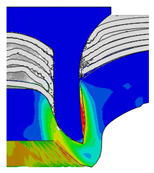

**Table 8:** Comparison of joint’s sectional deformation modes with different $D_{r}$ and $R_{L}$.

Rivet Length ($R_{L}$)/mm	Die Radius ($D_{r}$)/mm				
	0.50	0.75	1.00	1.25	1.50
**4.75**	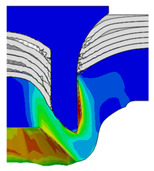	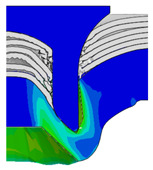	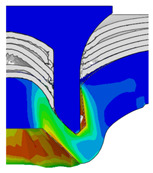	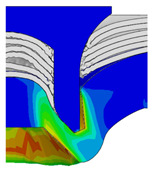	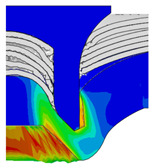
**5.00**	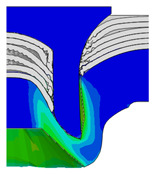	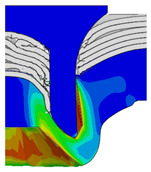	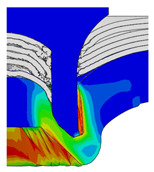	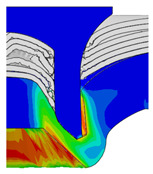	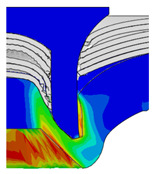
**5.25**	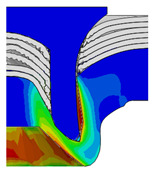	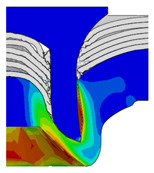	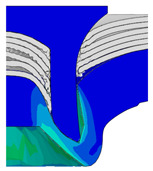	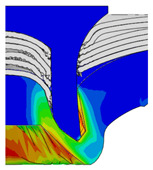	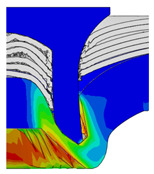

**Table 9:** Design factors and corresponding levels.

Levels	Symbols	1	2	3	4
**Rivet length (mm)**	$R_{L}$	5.00	5.25	5.50	5.75
**Die depth (mm)**	$D_{d}$	1.75	2.00	2.25	2.50
**Die gap (mm)**	$D_{g}$	0.50	0.75	1.00	1.25
**Die radius (mm)**	$D_{r}$	4.75	5.00	5.25	5.50

**Table 10:** Experimental results and corresponding *SNR*s.

No.	Taguchi Experimental Design				Experimental Results			
	$R_{L}$ (mm)	$D_{d}$ (mm)	$D_{g}$ (mm)	$D_{r}$ (mm)	BT ($1\times 10^{-2}\;mm$)	*SNR*1	IV ($1\times 10^{-2}\;mm$)	*SNR*2
**1**	5.00	1.75	0.50	4.75	50.163	34.008	26.963	28.615
**2**	5.00	2.00	0.75	5.00	69.718	36.867	22.146	26.906
**3**	5.00	2.25	1.00	5.25	87.086	38.799	17.322	24.772
**4**	5.00	2.50	1.25	5.50	110.477	40.865	14.648	23.316
**5**	5.25	1.75	0.75	5.25	47.342	33.505	44.264	32.921
**6**	5.25	2.00	0.50	5.50	58.026	35.272	30.018	29.548
**7**	5.25	2.25	1.25	4.75	73.611	37.339	24.067	27.628
**8**	5.25	2.50	1.00	5.00	97.048	39.740	18.830	25.497
**9**	5.50	1.75	1.00	5.50	36.565	31.261	45.530	33.166
**10**	5.50	2.00	1.25	5.25	44.266	32.921	35.610	31.031
**11**	5.50	2.25	0.50	5.00	60.739	35.669	31.555	29.981
**12**	5.50	2.50	0.75	4.75	85.675	38.657	27.067	28.649
**13**	5.75	1.75	1.25	5.00	25.501	28.131	50.899	34.134
**14**	5.75	2.00	1.00	4.75	34.126	30.662	44.407	32.949
**15**	5.75	2.25	0.75	5.50	51.294	34.201	40.207	32.086
**16**	5.75	2.50	0.50	5.25	74.799	37.478	38.846	31.787

**Table 11:** Response table for *SNR*1 of BT.

Means	Level	$R_{L}$	$D_{d}$	$D_{g}$	$D_{r}$
*SNR*1 (LTB)	1	**37.63**	31.73	35.61	35.17
	2	36.46	33.93	**35.81**	35.1
	3	34.63	36.5	35.12	**35.68**
	4	32.62	**39.19**	34.81	35.4
	Delta	5.02	7.46	0.99	0.57
	Rank	2	1	3	4
	Total mean	35.34			

**Table 12:** Response table for *SNR*2 of IV.

Means	Level	$R_{L}$	$D_{d}$	$D_{g}$	$D_{r}$
*SNR*2 (LTB)	1	25.9	**32.21**	29.98	29.46
	2	28.9	30.11	**30.14**	29.13
	3	30.71	28.62	29.1	**30.13**
	4	**32.74**	27.31	29.03	29.53
	Delta	6.84	4.9	1.11	1
	Rank	1	2	3	4
	Total mean	29.56			

**Table 13:** Weight values and relative closeness coefficient (RCC) obtained by Taguchi–TOPSIS method.

No.	Experiment Levels				Normalization of *SNR* Values ($z_{ij}$)		Weighted Values ($u_{ij}$)		Relative Closeness Coefficient (RCC) (LTB)	Rank
	$R_{L}$	$D_{d}$	$D_{g}$	$D_{r}$	***SNR*1 (LTB)**	***SNR*2 (LTB)**	***SNR*1 (LTB)**	***SNR*2 (LTB)**		
1	1	1	1	1	0.239	0.241	0.1070	0.1332	0.479	16
2	1	2	2	2	0.260	0.226	0.1160	0.1252	0.761	11
3	1	3	3	3	0.273	0.208	0.1220	0.1153	0.730	13
4	1	4	4	4	0.288	0.196	0.1285	0.1085	0.714	14
5	2	1	2	3	0.236	0.277	0.1054	0.1532	0.824	3
6	2	2	1	4	0.248	0.248	0.1109	0.1375	0.802	6
7	2	3	4	1	0.263	0.232	0.1174	0.1286	0.783	8
8	2	4	3	2	0.280	0.214	0.1250	0.1186	0.752	12
9	3	1	3	4	0.220	0.279	0.0983	0.1543	0.776	9
10	3	2	4	3	0.232	0.261	0.1035	0.1444	0.787	7
11	3	3	1	2	0.251	0.252	0.1122	0.1395	0.818	5
12	3	4	2	1	0.272	0.241	0.1216	0.1333	0.821	4
13	4	1	4	2	0.198	0.287	0.0885	0.1588	0.712	15
14	4	2	3	1	0.216	0.277	0.0964	0.1533	0.761	10
15	4	3	2	4	0.241	0.270	0.1076	0.1493	0.830	2
16	4	4	1	3	0.264	0.267	0.1179	0.1479	0.888	1

**Table 14:** Response table for RCC.

Means	Level	$R_{L}$	$D_{d}$	$D_{g}$	$D_{r}$
RCC (LTB)	1	0.671	0.698	0.747	0.711
	2	0.790	0.778	**0.809**	0.761
	3	**0.801**	0.790	0.755	**0.808**
	4	0.798	**0.794**	0.749	0.781
	Delta	0.130	0.096	0.062	0.097
	Rank	1	3	4	2
	Total mean	0.765			

**Table 15:** Comparison of joint’s forming quality indicators.

	BT (mm)	IV (mm)
**Initial design**	0.707	0.199
**Optimized design**	0.779	0.267
**Improvement rate**	10.18%	34.17%

## Data Availability

The original contributions presented in this study are included in the article. Further inquiries can be directed to the corresponding author.
